# System reliability analysis of the scoliosis disorder

**DOI:** 10.1186/s12891-020-03230-4

**Published:** 2020-03-31

**Authors:** Fatemeh Nouri, S. Hooman Ghasemi, Ji Yun Lee

**Affiliations:** 1 Department of Civil Engineering, Qazvin Branch, Islamic Azad University, Qazvin, 14778-93855 Iran; 2 Department of Civil and Environmental Engineering, Washington State University, Pullman, USA

**Keywords:** Target reliability, Scoliosis, Vertebral column, System reliability, Statistical parameters

## Abstract

**Background:**

Scoliosis is a spine abnormal deviation, which is an idiopathic disorder among children and adolescents. As a matter of the fact, distribution of loads on the patient’s spine and load-carrying capacity of the vertebral column are both random variables. Therefore, the probabilistic approach may consider as a sophisticated method to deal with this problem.

**Method:**

Reliability analysis is a probabilistic-based approach to consider the uncertainties of load and resistance of the vertebral column. The main contribution of this paper is to compare the reliability level of a normal and scoliosis spinal. To do so, the numerical analyses associated with the inherent random parameters of bones and applied load are performed. Then, the reliability indices for all vertebrae and discs are determined. Accordingly, as the main innovation of this paper, the system reliability indices of the spinal column for both normal and damaged backbone systems are represented.

**Results:**

Based on the required reliability index for normal spinal curvature the target system reliability level for scoliosis disorder is proposed.

**Conclusion:**

Since the proposed target reliability index is based on the strength limit state of the vertebral column, it can be considered as a reliability level for any proposed treatment approaches.

## Background

Scoliosis is a common disease that affects many children and adolescents. In a simple definition, scoliosis is a one-sided bending of the spine. Scoliosis is a serious malformation in which the spine abnormally changes with spinal rotation in three directions [26]. This abnormality progresses throughout growth, and the asymmetric loads due to spinal deformation cause more deformation, resulting in increased asymmetric loads, and this cycle continues [31]. In most cases, the growth of the spine has been observed in pre-puberty [28]. Spinal deformation in the idiopathic scoliosis abnormality is generally described as the lateral deformity caused by the lateral spine curvature [30]. The deformity caused by the spine in this disorder involves deformation and displacement in three directions and the spinal axis rotation [17]. The effective treatment for the curvature of the spine is to install the rod and curvature correction by loading force. The rod that is mounted on the spine is responsible for bearing the forces created by the spine and skeletal deformation. For this reason, it is very important to estimate the forces needed to be loaded on the rod used in scoliosis curvature repair in a way that does not cause bone failure [26]. Measuring the forces involved in the spine in a living tissue environment is difficult, and numerous studies have been done to measure the distribution of force on the spine and this information is available. In 1989, Stokes examined the spine of 40 patients with idiopathic scoliosis in adults to examine the relationship between the vertebral spinal cord and deviant and lateral curvature in the spinal column in scoliosis and indicated that spinal rotation had a close relationship with deviation. Then, Stokes defined the relationship between the vertebral rotation and lateral curvature in the spine. In 2011, Shi et al. examined the association between the progression of idiopathic anomalies in adults and the anomalous development of the anterior part of the spine and indicated that the rate of growth stimulated the progression and increased risk of scoliosis. Salmingo et al. [26] presented a force method based on a finite element analysis to estimate spinal force inputs in a living tissue environment by examining the shape deformation used in the treatment of scoliosis abnormality curvature correction using 3D imaging. They showed that bending stresses depend directly on the curvature angle on the deformed rod. In 2013, Salmingo et al. measured the amount of bar deformation used in the treatment of surgery before and after treatment. They found the correlation between the intensity of the force and the angle of correction by measuring the applied force. Little et al. [19] tried to find out how deformation was affected by the severity of corrective forces to predict the severity of the tension required to treat spinal curvature in scoliosis abnormalities. Their research showed that there is a direct relationship between the compressive forces of the connections used and the degree of curvature change. In 2015, Abe et al. analyzed the amount of force that is necessary to correct the spinal cord by limited finite element analyzes and examining 20 patients who underwent spinal cord correction surgery between 2009 and 2011. Schlösser et al. [27] examined the relationship between the three-dimensional displacements of the vertebrae. In 2015, Cheuk et al. evaluated the mechanical properties of the spinal bones using finite element analysis.

However, the applied load and the structural resistance of the backbone system are both random variables which involved the uncertainties. Therefore, there is a need for a probabilistic approach to estimate the load-carrying capacity of the scoliosis disorder. To do so, in this study, first, it is attempted to collect the inherent uncertainties parameters impacting on the load-carrying capacity of the vertebral column. Then, the reliability index of each disc and vertebra is calculated using the reliability analysis. Then, the system reliability index of the backbone is represented for both normal and abnormal vertebral columns. The obtained system reliability level of the normal vertebral column is expected to be considered as target system reliability. Accordingly, the abnormal backbone particularly the scoliosis one can be cured using any proposed method including the surgery to retrieve the reliability level to the normal one.

## Methodology

To determine the safety level of the vertebral column systems, there is a need to consider the probabilistic-based approaches. The reliability index is a world-widely measure to evaluate the safety level of a component or a system with consideration of load and resistance distributions. The main step in reliability analysis is to determine the Limit State Function, which can be constructed based on the expected performance level of the structure. In general, several different limit state functions have been considered for structures including strength, service, fatigue, and extreme events. In this present study, to investigate the resistance-ability of the backbone system due to the normal load, the strength limit state function is considered. Therefore, first, the structural component and the statistical parameters of loading and resistance associated with their distributions for normal backbones and scoliosis ones should be determined. Then, the reliability level of each structural component is computed using Monte Carlo simulation. Finally, the reliability indices of both mention system (sound and damaged due to the scoliosis disorder) are calculated.

### Mechanical properties of vertebrae

This section intends to specify the main structural component of the backbone and their presented mechanical properties of those. As a structural point of view, vertebrae and intervertebral discs can be considered as two main structural components for axial load carrying capacity. Each vertebra is composed of cancellous bone (soft part) and cortical bone (the hard part). In this section, the mechanical properties of both cancellous and cortical bone are reviewed.

Many studies have been conducted to determine the mechanical parameters, including the density of the vertebrae [25]. Ebbesen et al. [3] measured different densities of vertebrae such as ash density of cortical and cancellous and bone mineral density. They showed the relationship between age, mass, and density of vertebrae. Also, Helgason et al. [12] studied the relationships between the physical and mechanical properties of bone using ash density. They utilized the proposed relationship between the ash and apparent density proposed by Keyak et al. [16]. Accordingly, [3] tested the yield stress of several vertebrae. In another study, [20] examined the relationship between ash density and maximum stress. Kopperdahl and Keaveny [15] researched to evaluate the stress-strain behavior for the cortical part of vertebrae. Also, Öhman et al. [22] conducted a real test to investigate the mechanical properties of bone. Patel et al. [23] used the Hounsfield unit to predict the stress limit of the bone for cortical tissue. Recently, Azari et al. [2] collected the mechanical properties of vertebrae. As can be seen, based on the reported literature, the mechanical properties of the bone behave as a random variable. Therefore, in this study, although the moduli of elasticity of bones are considered as the deterministic parameters (Table 1), the yield stresses of vertebrae are assumed to be treated as random normal variables (Table 2).

**Table 1:** Assumed moduli of the vertebra

	Modulus of elasticity (MPa.)	Poisson’s ratio
Cortical bone	E_xx_ = 11,300	v_xy_ = 0.484
	E_yy_ = 11,300	
	E_zz_ = 22,000	v_yz_ = 0.203
	G_xy_ = 3800	
	G_yz_ = 5400	v_xz_ = 0.203
	G_xz_ = 5400	
Cancellous bone	E_xx_ = 140	v_xy_ = 0.450
	E_yy_ = 140	
	E_zz_ = 200	v_yz_ = 0.315
	G_xy_ = 48.3	
	G_yz_ = 48.3	v_xz_ = 0.250
	G_xz_ = 48.3

**Table 2:** Statistical parameters of yield stress for vertebrae

Tension		Compression	
Mean (MPa.)	STD	Mean (MPa.)	STD
Yield Stress – Cortical			
75	13.98	176	28.4
Yield Stress - Cancellous			
1.9	0.86	1.78	0.58

where E_ij_ and G_ij_ denote the modulus of elasticity in different directions. And v_ij_ represents the Poisson’s ratio of bone in various directions.

### Mechanical properties of intervertebral discs

Intervertebral discs with exerting the flexibility and small movements between the adjacent vertebrae make it possible to bend the entire spine. Intervertebral disc also distributes the load evenly on the body of the vertebrae. The intervertebral discs are extremely resistant under pressure and are very effective in absorbing shock in the spin. The intervertebral discs have three main layers [1]: 1- core layer which is called “nucleus pulposus”, 2- the intermediate layer which is named “annulus fibrosus”, and 3- outer thin layer known as cartilage endplate. The high amount of water in disc causes hydrostatic pressure and viscous-elasticity behavior. Several studies have been conducted to determine the mechanical properties of discs [13, 14]. Furthermore, Pollintine et al. [24] studied the time-dependent changes in the time-spatial shape of the discs and vertebrae in the spinal column. However, Wang et al. [32] used a 3D viscoelastic verified finite element model to investigate the mechanical properties of the L2-L3 lumbar vertebrae. Larde et al. [18] studied 36 cases of bone marrow infection in the spine for 3 years. The mean age of the patients was 42 ± 5 and in the range of 10 to 72 years. In healthy subjects, the CT scan obtained from discs between 73 ± 13 units of Hounsfield on a 1000HU scale. Wintermantel et al. [33] investigated the condition of 34 patients with lumbar disc herniation the Hounsfield’s unite for a healthy part of the disc has been reported between 70 and 80 units. To measure the tensile strength of the intervertebral discs, Adams [1] conducted an experimental study. Table 3 shows the tensile properties of annulus fibrosus, derived from uniaxial tension tests on small annulus samples from human lumbar discs aged 48–91 years. Adams performed preliminary tests indicating that the tensile stress in the intervertebral disc core is about 0.26 MPa (range 0.08–0.64). Besides, Mow and Huiskes [21] stated that the nature of annulus fibrosis is resistant to tension and elongation at the disc caused by the movement of the adjacent vertebrae and the pressure of the inflammation. Galante [4] measured the tensile properties of annulus fibrosis and showed that the function of this tissue is a nonlinear, heterogeneous, and anisotropic and it is viscose material and its properties are sensitive to its hydration state. Skaggs et al. [29] examined the tensile properties of single-layer samples along the dominant fiber direction, and obtained E values from 60 to 140 MPa, depending on the region of the annulus. Young’s modulus was obtained for multilayer samples of 25 MPa in a radial direction of less than 0.5 MPa. The values measured for the Poisson ratio were significantly greater than 0.5, which indicates the anisotropic behavior of the annulus area. Mow and Huiskes [21] collected and presented the results of various experiments in the biomechanics of the base of orthopedics and biomechanics, including the intraocular properties and mechanical behavior of the discs in swelling, elasticity, pressure and cutting. Also, in this study, the tensile modulus of internal and external parts of the annulus fibrosus have been presented taken by and Iatridis [14]. in this study, it is attempted to model both annulus and nucleus tissue of disc. Therefore, based on the discussed precious studies in this section, the main mechanical properties of disc including shear relaxation modulus, bulk relaxation modulus, and relaxation time constant are assumed as the deterministic parameters (see Table 3). However, to perform the reliability analysis, the yield stresses of annulus and nucleus part of the disc are considered as a random variable, which represented in Table 4.

**Table 3:** Mechanical properties of disc

The Material Constants of Annulus Matrix and Nucleus Pulposus Using the Prony Series			
Relaxation of	Shear Relaxation Modulus (MPa.)	Bulk Relaxation Modulus (MPa.)	Relaxation Time Constant (second)
Annulus matrix, E = 8.0 (MPa.), ν = 0.45	g_1_ = 0.399	k_1_ = 0.399	τ_1_ = 3.45
	g_2_ = 0.000	k_2_ = 0.300	τ_2_ = 100
	g_3_ = 0.361	k_3_ = 0.149	τ_3_ = 1000
	g_4_ = 0.108	k_4_ = 0.150	τ_4_ = 5000
Nucleus pulposus, E = 2.0 (MPa.), ν = 0.49	g_1_ = 0.638	k_1_ = 0.0	τ_1_ = 0.141
	g_2_ = 0.156	k_2_ = 0.0	τ_2_ = 2.21
	g_3_ = 0.120	k_3_ = 0. 0	τ_3_ = 39.9
	g_4_ = 0.0383	k_4_ = 0.0	τ_4_ = 266
	g_5_ = 0	k_4_ = 0.0	τ_4_ = 500

**Table 4:** Statistical parameters of yield stress for disc

Tension		Compression	
Mean (MPa.)	STD	Mean (MPa.)	STD
7.30	2.30	3.65	1.15
Yield Stress - Nucleus			
Tension		Compression	
Mean (MPa.)	STD	Mean (MPa.)	STD
0.260	0.0819	0.260	0.0819

## Structural analysis

### Geometry modeling

**Fig. 1:**
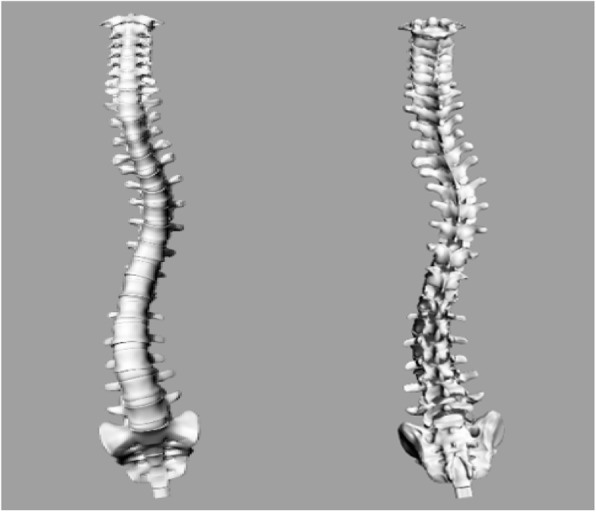
The CT scan for considered vertebral column and its 3D model

For modeling, a 3D model of the healthy spine was developed using Rino software. Then, the cancellous and cortical part of the vertebrae simulated according to the collected data. Also, for modeling intervertebral discs, annulus fibrosus was simulated as a layered cylinder and nucleus pulposus simulated as a cylinder. For modeling of ligaments and tendons, the spring replacement model was used. First, a healthy three-dimensional spinal column model was developed. A CT scan of a backbone with scoliosis developed with EOS technology was used for 3D modeling (see Fig. 1).

**Fig. 2:**
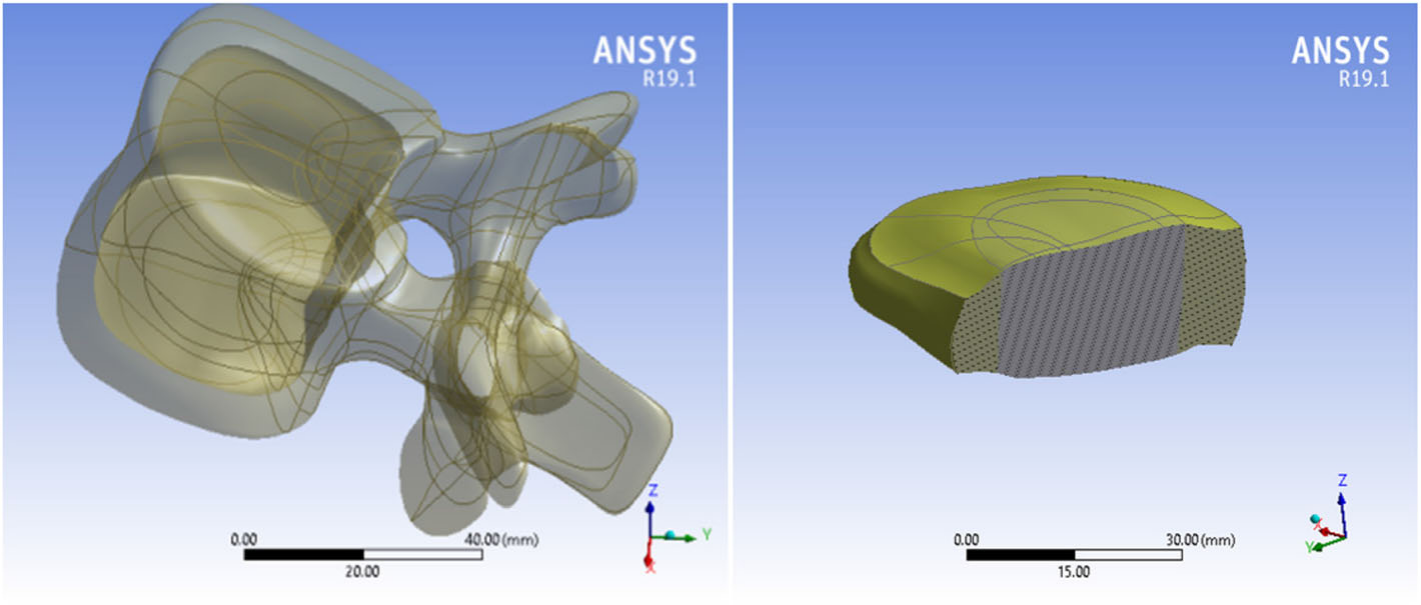
3D structural modeling of vertebra and disc using ANSYS

The amount of displacement of each vertebra was measured from the CT scan image and applied to a healthy sample model, and the patient’s 3D model was prepared. The vertebra is composed of two parts of cortical and cancellous. The intervertebral discs consist of annulus and nucleus as viscoelastic material (see Fig. 2).

**Fig. 3:**
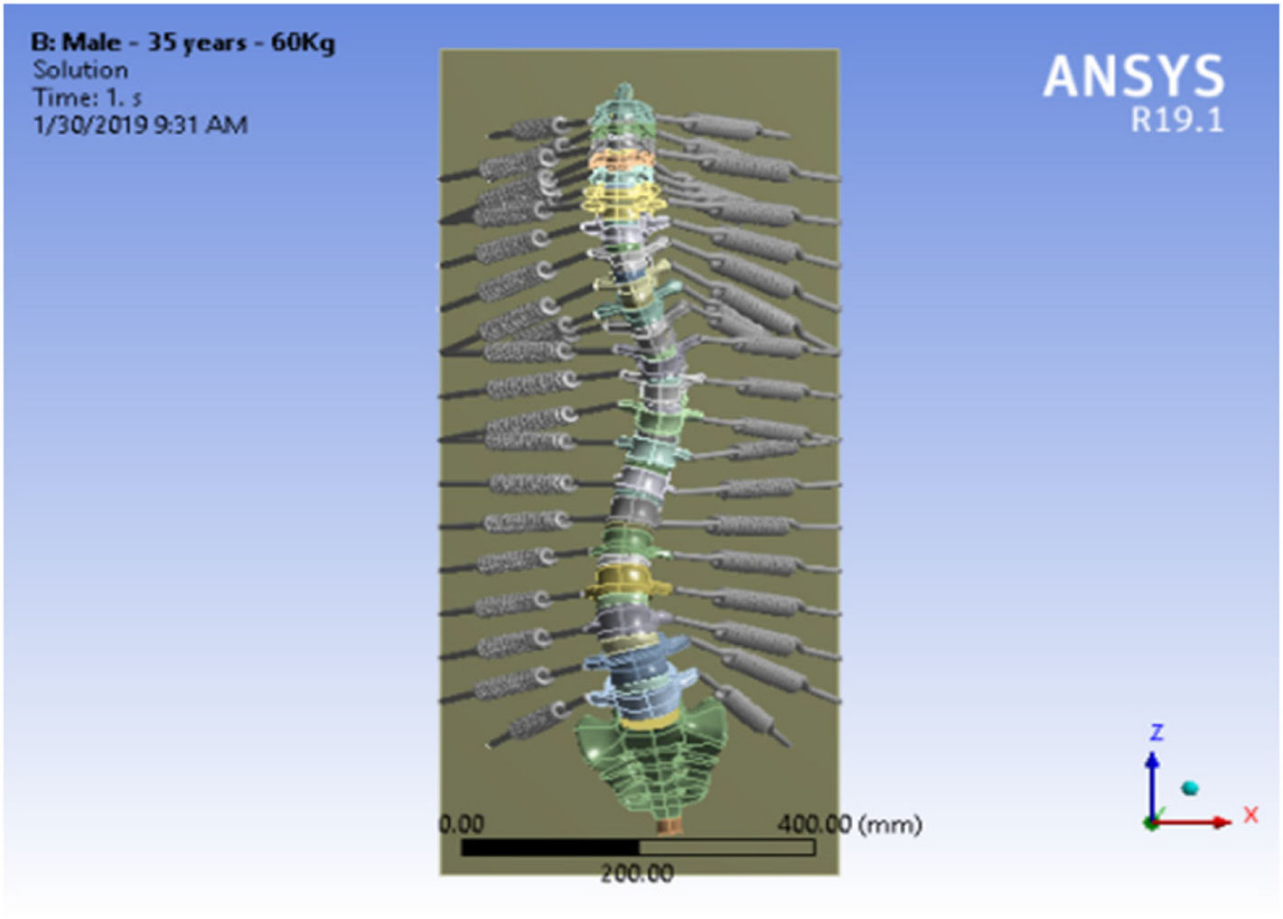
Full vertebral column modeling associated with considered ligaments using linear springs

In this research, the ligaments and tendons were also modeled using linear springs with stiffness equal to 205.6 N/mm (see Fig. 3).

Also, the scoliosis model of the spine developed from normal spine and data getting from by EOS scans and is simulated using the information obtained from the scanned image and prepared sample of the healthy spine. The 3D model used in the Ansys. To perform the reliability analysis, there is a need to generate a sufficient number of models. To reach this intention, 24 samples, 12 female samples (20 years old 30 kg and 40 kg, 35 years old weighing 50 kg, 60 kg, 70 kg, and 80 kg) and 12 male samples (20 years old 30 kg and 40 kg, 35 years old weighing 50 kg, 60 kg, 70 kg and 80 kg) for a healthy case and scoliosis case are generated using ANSYS Workbench software.

### Loads and supports

**Fig. 4:**
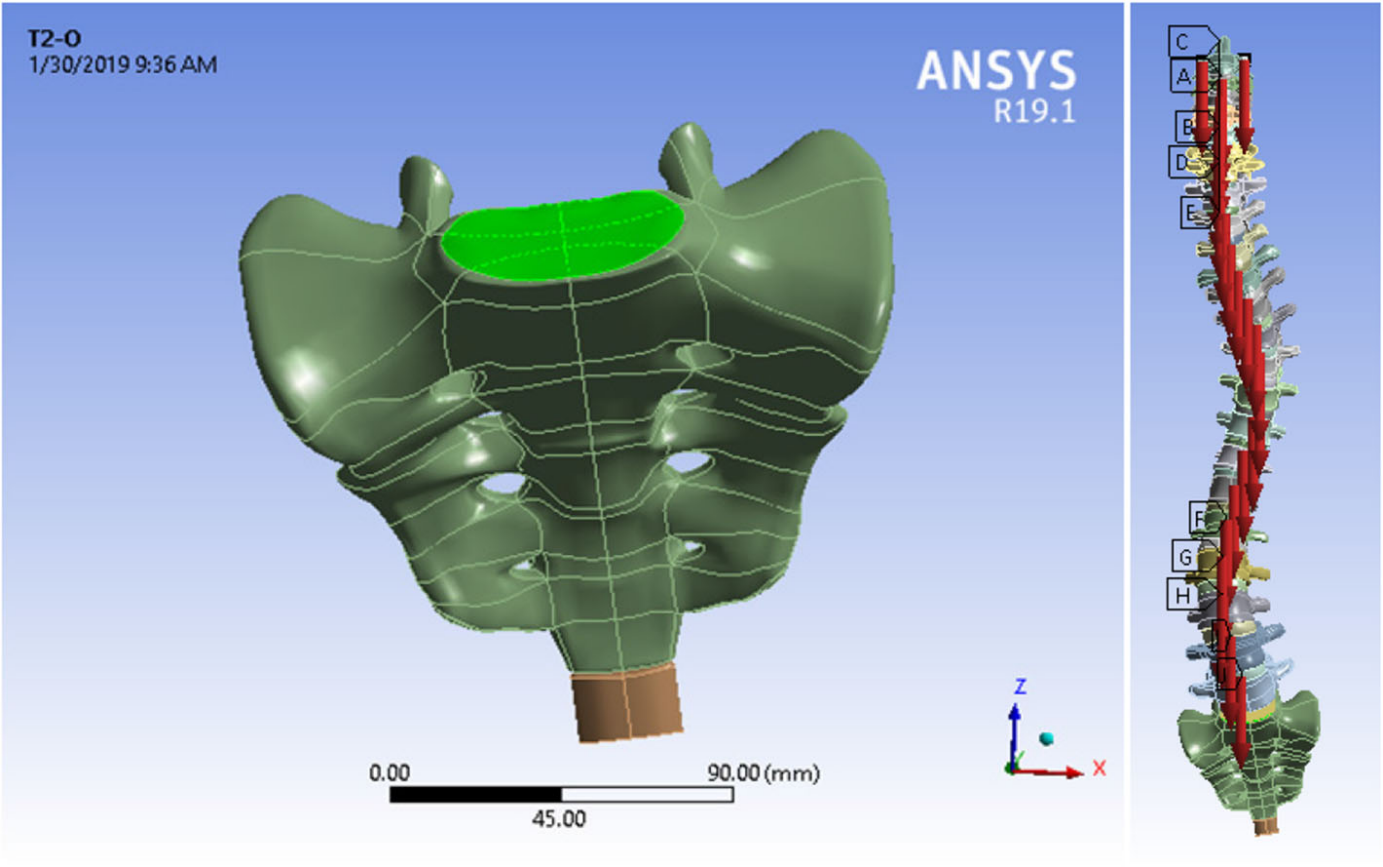
Loading and boundary conditions considered for vertebral column analysis

For loading, assuming that about 50–60% of the body’s weight is tolerated by the spine, this amount divided between the vertebrae evenly. Weight of head assumed about 4.5–5.5 kg, which is represented by two concentrated symmetrical forces on the second cervical vertebra, the C2. Considering the case in a standing position, the upper surface of the Sacrum was defined as fixed support. Thus, the displacement of the sacrum was limited in all directions (see Fig. 4).

### Validation

**Fig. 5:**
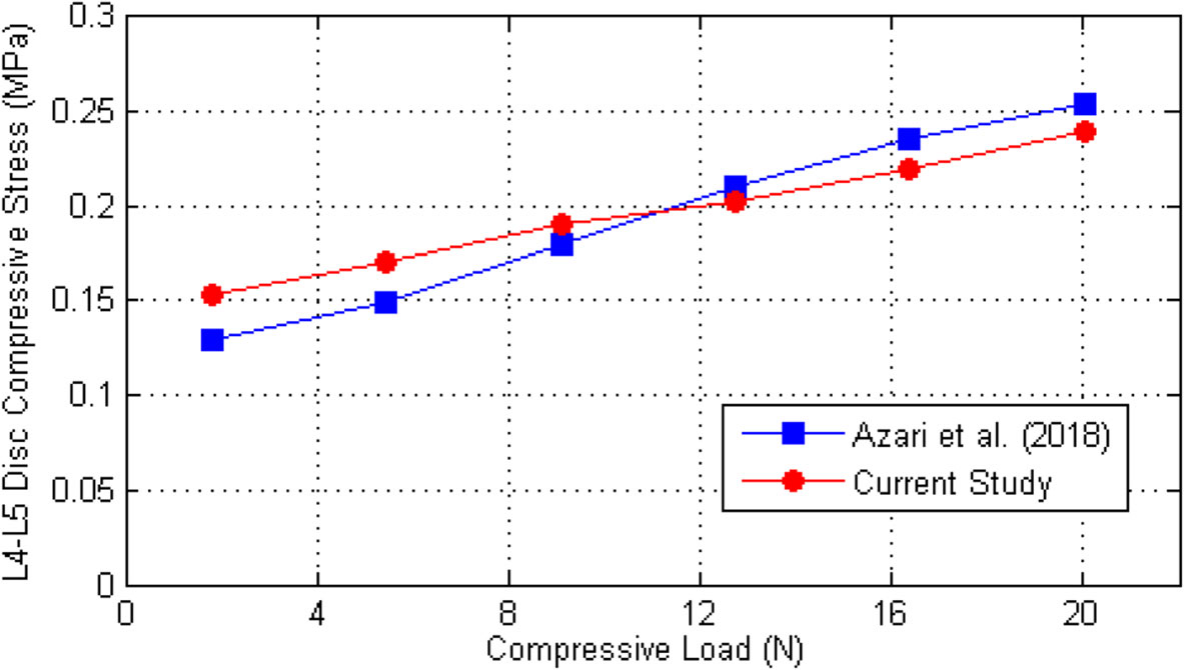
Verification of the FEM results corresponding to the stress state of L4-L5 Disc subjected to the compression

To assess the accuracy of the obtained result, the stresses state of the disc between the 4 and 5 lumbar vertebrae in a standing position subjected to the compression was compared with the presented result of Azari et al. [2]. As can be seen in Fig. 5, the obtained stress results in the current study were reached to almost the same result presented by Azari et al. [2], which can be considered as a verification of the currently conducted research.

## System reliability

### Failure mechanics

The vertebral column consists of the two main structural components which may fail in different manners. In this study, the tension and compression principal stress states of cortical and cancellous are compared with the yield stress limit to figure out the failure mechanism of vertebrae. Accordingly, the vertebral probabilities of failure are determined based on the parallel probability of failure of both cortical and cancellous either corresponding to the tension yielding limit or compression yielding limit states. The same scenario may happen for intervertebral discs. It means both tension and compression stress states within annulus and nucleus are compared with the yielding tolerance of annulus and nucleus to specify the failure probability of intervertebral discs. Eventfully, the vertebral system failure probability is consists of a series model of vertebrae and intervertebral discs.

### Reliability analysis

Here in this section, based on the obtained stresses results of the vertebral columns, the reliability index for each component of the spine was calculated. As it was mentioned, to perform the reliability analysis, there is a need for the establishment of the limit state function. There are several limit states associated with the various structural performance concerned [5–10]. In this study, the strength limit state function is considered as the backbone performance function. Therefore, the reliability indices for both cortical and cancellous of vertebra tissues, and both tissues of intervertebral discs (annulus and nucleus) are computed. In general, the reliability indices can be determined using the distributions of the structural resistance (R) and applied load (Q). If the limit state function is formulated as a linear function and the load and resistance distributions follow the normal one, the reliability index can be computed using the following equation.

1 $$ \beta =\frac{\mu_R-{\mu}_Q}{\sqrt{\sigma_R^2+{\sigma}_Q^2}} $$

where *μ*_*R*_ and *μ*_*Q*_ present the mean value of load and resistance. Also, *σ*_*R*_ and *σ*_*Q*_ denote the standard deviation of load and resistance. However, the establishment of the limit state function requires a closed-form equation. In this study, as a-state-of-the-art, a novel procedure is utilized to determine the reliability index. In this innovative procedure, first, the stress’s state of all elements is ascertained using finite element analysis. Then, the most vulnerable zones of the vertebral column due to the axial loading conditions are recognized. Accordingly, several FEM models of the vertebral column are created based on the random variables of the material and geometry. Finally, based on the obtained random stress results, the distribution of the applied loads are determined. On the other hand, based on the collected data of the stress limit of backbone components the resistance distribution of vertebrae (cortical and cancellous) and intervertebral discs (annulus and nucleus) are generated. Hence, the reliability index of each component is determined.

Finally, the system reliability indices of the normal and scoliosis backbones are determined. To capture this intention, the system reliability formulation is constructed with consideration of the parallel and series algorithm for the given backbone. Since the applied forces on the vertebrae are distributed between cortical and cancellous, cortical and cancellous within the vertebrae are constituted a parallel system. The same condition can be observed for intervertebral discs, in which both tissues of annulus and nucleus construct the parallel system. The failure of the parallel systems can be derived using the following equation:

2 $$ {P}_F={\varPi P}_{Fi} $$

where *P*_*f*_ is the probability of the system failure, and *P*_*fi*_ is the failure probability of the i^th^ component. However, the load contribution between the vertebrae and discs can be assumed as the series system. The governing equation to describe the failure probability of a series system can be written as follow:

3 $$ {P}_F=1-\varPi\ \left(1-{P}_{Fi}\right) $$

For considered structural system (vertebral column), four strength limit state functions are defined as follows

4 $$ \left\{\begin{array}{c}{g}_{Annlus}={R}_{Annlus}-{Q}_{Annalus}\ \\ {}{g}_{Nucleus}={R}_{Nucleus}-{Q}_{Nucleus}\ \\ {}\begin{array}{c}{g}_{Cancellous}={R}_{Cancellous}-{Q}_{Cancellous}\\ {}{g}_{Cortical}={R}_{Cortical}-{Q}_{Cortical}\ \end{array}\end{array}\right. $$

where *g*_*i*_, *R*_*i*_, and *Q*_*i*_ are limit state function, PDF of ultimate resistance, and PDF of applied load for the considered vertebral column sections. Thus, the failure probability (*P*_*Fi*_) of each section is derived using any conventional probability analysis. Accordingly, since the loading path is distributed between the annulus and nucleus, the failure probability of the discs (*P*_*FDisc*_) can be considered as the series system can be formulated as shown in Eq. 5.

5 $$ {P_F}_{Disc}=\left({P_F}_{Annlus}\right)\left({P_F}_{Nucleus}\right) $$

Similarly, the load path is distributed between cortical ad cancellous, therefore, the failure probability of vertebra (*P*_*FVertebra*_) can be defined as follows

6 $$ {P_F}_{Disc}=\left({P_F}_{\mathrm{cancellous}}\right)\left({P_F}_{\mathrm{cortical}}\right) $$

It is worth mentioning that failure of either vertebra or disc leads to the system failure, therefore, the sectional failure probability can be considered as a series system and is extracted from Eq. 7.

7 $$ {P_F}_{sectional}=1-\prod \left(1-{P}_{Disc}\right)\left(1-{P}_{Vertebra}\right) $$

Eventually, all vertebra in a backbone chain is connected as a series system. Therefore, the probability of the backbone is defined using Eq. 8.

8 $$ {P_F}_{backbne}=1-{\prod}_{i=1}^{n=33}\left(1-{P_F}_i\right) $$

where *P*_*Fbackbne*_ is the failure probability of the entire backbone system and *i* denotes the index of the vertebra and its associated disc number.

**Fig. 6:**
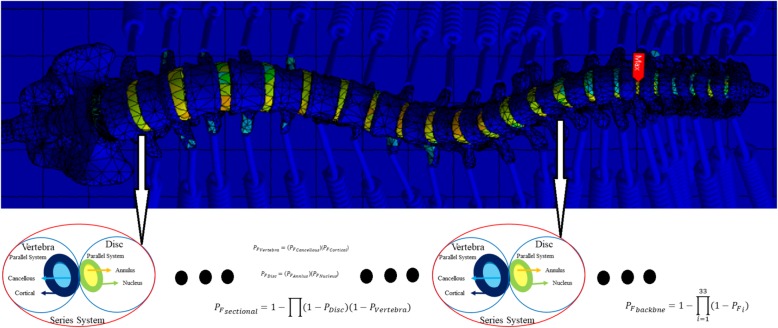
The proposed framework to determine the system reliability for the vertebral column

Figure 6 shows the schematic producer which have to be accomplished to determine the reliability index of the vertebral column.

## Results

In this section, several FE models with the generated random properties were analyzed to compute the stress states of the vertebral column. The most vulnerable zones of the normal vertebral column for different human weights are tabulated in Table 5.

**Table 5:** The most vulnerable zone of normal vertebral column for different human weights

Normal					
Maximum Stress					
Weight (KG)		Cortical	Cancellous	Annulus	Nucleus
30	Tension	T2	C7	C7	T6
	Compression	C7	C7	C6	C7
40	Tension	T2	C7	C7	T6
	Compression	C7	L3-C7	C6	C7
50	Tension	T2	C7	C7	T6
	Compression	C7	L3-C7	C6	C7
60	Tension	T2	C7	C7	T6
	Compression	C7	L3-C7	C6	C7
70	Tension	T2	C7	C7	T6
	Compression	C7	L3-C7	C6	C7
80	Tension	T2	C7	C7	T6
	Compression	C7	L3-C7	C6	C7

As it was observed, the most vulnerable stress zone for normal backbone are placed on discs C5-C6-C7 or T6-T7-T8 and vertebrae C7, T2, or L3 vary depending on human body weights. Also, Table 6 represents the most vulnerable zone of the vertebral column associated with the different human weights for one of the considered scoliosis curvatures.

**Table 6:** The most vulnerable zone of scoliosis vertebral column for different human weights

Scoliosis					
Maximum Stress					
Weight (KG)		Cortical	Cancellous	Annulus	Nucleus
30	Tension	T1	T7	C7	T7
	Compression	T1	T10	C6	T10
40	Tension	T1	T7	C7	T7
	Compression	C7	T10	C6	T10
50	Tension	C7	T7	C7	C7
	Compression	C7	T10	C6	C7
60	Tension	C7	T7	C7	C6
	Compression	C7	T10	C6	C6
70	Tension	C7	T8	C7	C6
	Compression	C7	T10	C6	C6
80	Tension	C7	T8	C7	C6
	Compression	C7	T10	C6	C7

Based on the obtained results, the vulnerable stress zones of the considered scoliosis spinal column are placed on discs T6-T7or C7 and verte1brae T1, T8, T10. Based on several FEM performed model in this study, although the most vulnerable zone of scoliosis vertebral column varies depending on the curvature, it was observed that the maximum stress states are approximately concentrated on the inflection points of the scoliosis curvature (See Fig. 6).

**Fig. 7:**
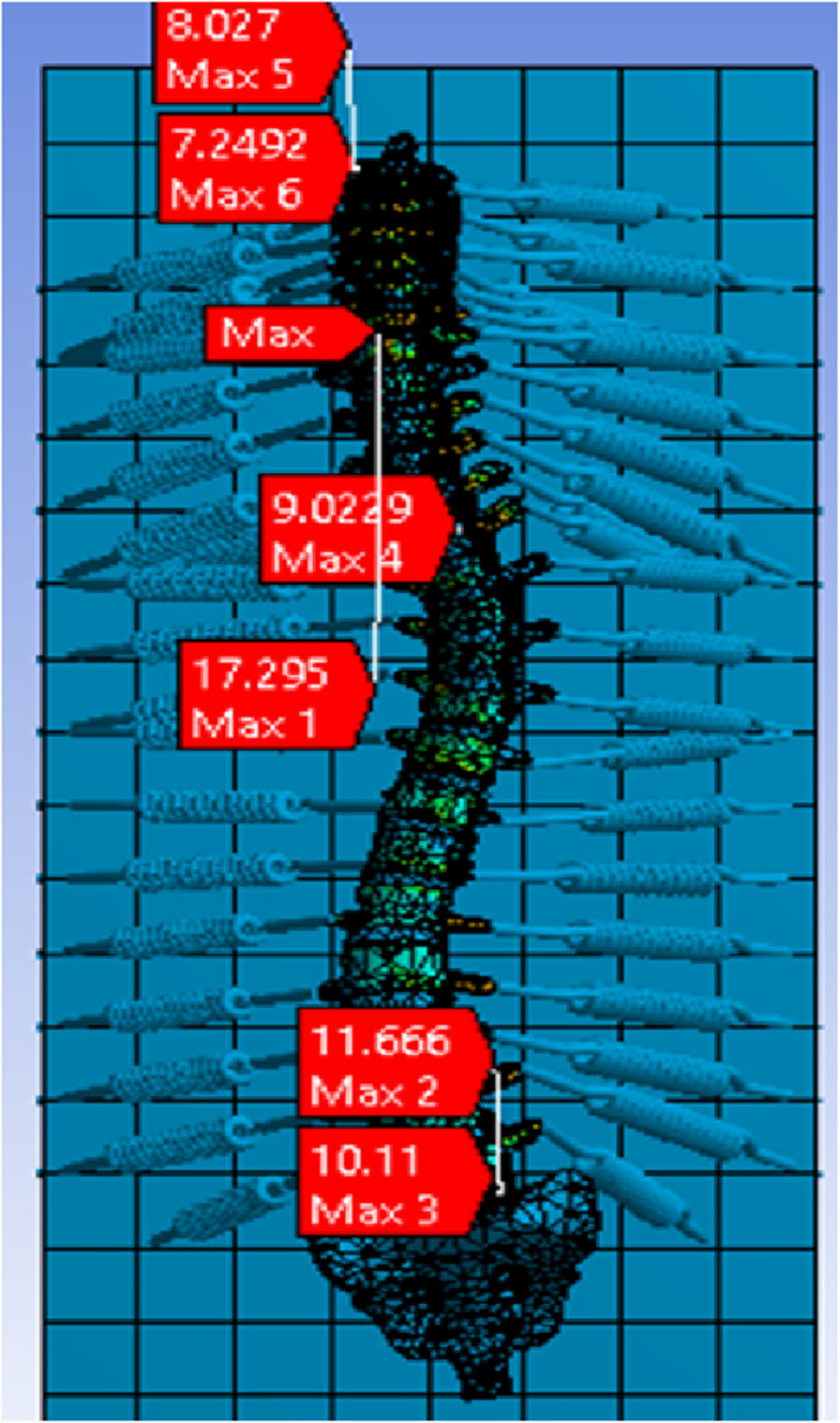
Vulnerability stress analysis of vertebral column for a case study (Scoliosis, Female, 30 KG)

As shown in Fig. 7, the maximum stresses were observed near the inflection points of the spinal curvature for a 30 Kg-female-case-study suffered the scoliosis disorder.

**Fig. 8:**
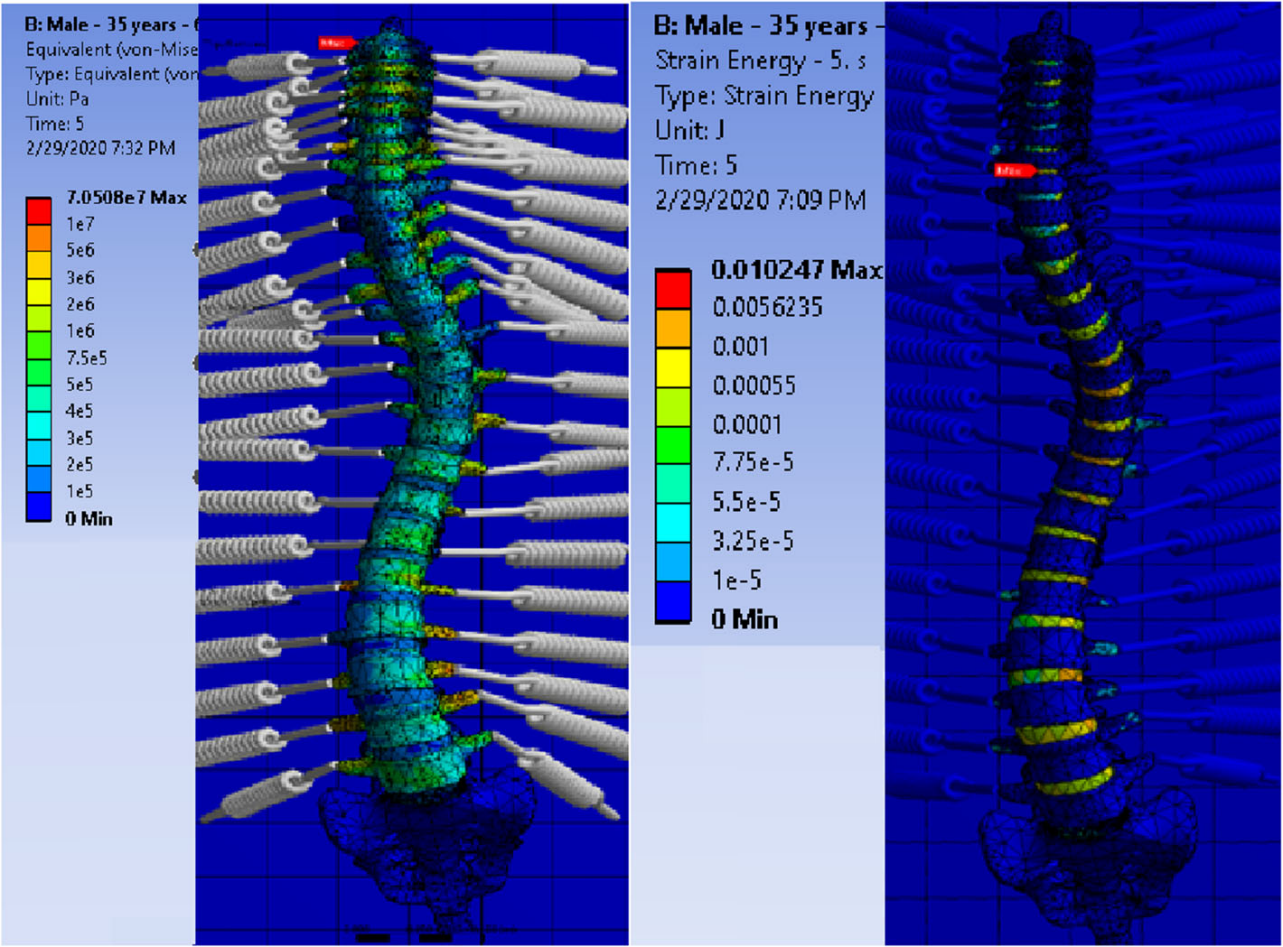
Stress distribution for a scoliosis vertebral column (left), and strain energy development throughout the scoliosis vertebral column (right)

As can be seen in Fig. 8, although the vertebrae tolerate major induced stress, the foremost strain energy carries by discs. Since disc made of the softer material and a low amount of stress magnitude can lead to the gigantic amount of strain. Indeed, the discs work like energy dampers to imped the failure of the vertebra. Therefore, the most vulnerable zones are discs. This fact was revealed based on the reliability analysis. The conducted reliability analysis of this research indicates that the failure probabilities of discs are significantly higher than the vertebrae.

Regarding the evaluated stresses of each element, the reliability indices for all components of the spine were obtained for both normal and scoliosis ones. The obtained result for each component was taken to the account to determine the reliability index of each vertebra and disc. Accordingly, the system reliability index was calculated. Table 7 represents the failure probability of the normal and scoliosis spin for the various human weights.

**Table 7:** Failure probability of the normal and scoliosis vertebral column subjected to the human weight

		30 KG	40 KG	50 KG	60 KG	70 KG	80 KG
Normal Spine	P_f_	2.05 × 10^−5^	2.57 × 10^−5^	3.75 × 10^−5^	4.93 × 10^−5^	6.59 × 10^−5^	8.82 × 10^−5^
Normal Spine	*β*	4.10	4.05	3.96	3.89	3.82	3.75
Scoliosis Spine	P_f_	2. 77 × 10^−4^	1.14 × 10^−3^	1.82 × 10^−3^	2.22 × 10^−3^	2.45 × 10^−3^	3.18 ×10^−3^
Scoliosis Spine	*β*	3.45	3.05	2.91	2.85	2.81	2.73

Also, the corresponding reliability indices are presented in Table 7 for both normal and scoliosis spines at the different body weights. It is worth mention that the Monte Carlo simulation was conducted to compute the failure probability of the given limit state function. Due to the enhancement of the asymmetrical loading effect, the differences in the system reliability indices for heavier weights are being more significant.

## Discussions

As mentioned earlier, scoliosis is a progressive process that is usually taken just before or during puberty, and it is more common in women than men. Depending on the age of the diagnosis, this disorder is divided into three categories: childhood, adolescence, and youth. Several types of scoliosis affect people. Until this time, the most common type is idiopathic. Scoliosis is, in fact, a complex deformity, and its description requires a three-dimensional examination of this abnormality. In only 20% -15 cases, the cause of the deformity is known, and in most cases, the cause is unknown, which has been called idiopathic scoliosis. The vertebral column, also called the backbone or spine, is the main part of the axial skeleton. The vertebral column is made of the series of bones known as “vertebrae” which are connected by intervertebral discs. Normally, there are 33 vertebrae (see Gray [11]) within the vertebral column. The upper part is made of 24 vertebrates and the lower part consists of nine bone located in both the sacrum and in the coccyx. There are seven cervical vertebrae, 12 thoracic vertebrae, and five lumbar vertebrae.

In this study, a new procedure was established to measure the safety level of the vertebral column using a proposed system reliability approach. The obtained reliability index divulges the deficiency level of a scoliosis vertebral column in comparison with a normal one, which helps us to determine the severity level of the disorder and consider a proper treatment to redeem the patient. The advantage of the reliability analysis for this problem is to utilize the resilience analysis to make a design for recovery.

As it was observed, the most vulnerable stress zone for normal backbone subjected to the self-weight loading are located on discs C5-C6-C7, T6-T7-T8, or T9-T10, and on vertebrae C6-C7, T2-T3, or L2-L3-L4. Although the most vulnerable zone of scoliosis vertebral column varies depending on the curvature, the maximum stress states are approximately concentrated on the inflection point of the curvature. Hence, it can be stated that the maximum stress vulnerable zone in normal cases has been distributed over different discs. However, for the scoliosis case, the disc C7 and C6 have been construed as the suspected zone for inconveniences. However, as can be seen, the interpreter of the results depends on many factors and it is hard to measure the severity of the scoliosis disorder. Therefore, there is a need for a new safety indicator that can represent a safety measure to evaluate the resilience condition of a vertebral column.

**Fig. 9:**
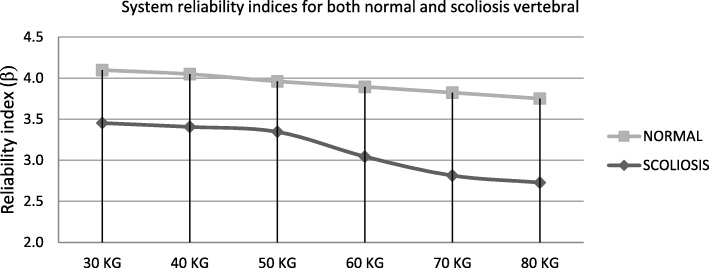
System reliability indices for both normal and scoliosis vertebral column with consideration of the different human body weights

Figure 9 compares the system reliability indices for both normal and scoliosis vertebral column. As can be seen, by increasing the overall weight of the human body the reliability index is decreased. Also, the main advantage of the probabilistic analysis is to determine the reduction capacity of the scoliosis disorder in terms of the reliability index. The shrinking of the reliability index can be considered as the resilience metric for patient therapy.

## Conclusions

The scoliosis disorder asymmetrically distributes the weight of the human body throughout the spinal column. Accordingly, the severe damages are observed corresponding to the asymmetrical load distribution. However, to treat the scoliosis disorder, the reliable stress states of the vertebral column shall be determined. Nonetheless, the statistical parameters of the spinal column showed that the applied load and load-carrying capacity are both random variables. In this study, first, it was attempted to collect statistical parameters of the load and mechanical properties of the structural components of the backbone. Accordingly, several FE models with the generated random properties were analyzed to compute the stress states of the vertebral column. Based on the obtained results, the vulnerable stress zone for both normal and scoliosis spinal column was determined. Accordingly, the reliability analysis was conducted to determine the reliability level of the structural component of the vertebral column including disc (for both Annulus and nucleus) and vertebra (for both cortical and cancellous). Finally, as the state-of-the-art, the reliability index of the whole system for both normal and scoliosis vertebral columns was calculated using Monte Carlo simulations. In this paper, the obtained system reliability for the normal vertebral column is proposed as a target reliability system. Eventually, the proposed target reliability can be considered as the appropriate measure for resilience analysis, which can help us to propose an appropriate treatment for the vertebral column to retrieve to the acceptable performance level. It should be noted that although the obtained reliability index for a normal vertebral column can be extended to all normal cases, the reliability analysis for a scoliosis disorder should be performed using a CT scan of an individual patient.

## Data Availability

The datasets used and/or analyzed during the current study are available from the first author on reasonable request.

## Abbreviations

*β*: Reliability index
E: Modulus of elasticity
g: Shear relaxation modulus
k: Bulk relaxation modulus
*μ*_*Q*_: Mean value of loading
*μ*_*R*_: Mean of resistance
ν: Poisson’s ration
P_f_: Probability of failure
Q: Random variable of loading
R: Random variable of resistance
*σ*_*Q*_: Standard deviation of loading
*σ*_*R*_: Standard deviation of resistance
τ: Relaxation constant
